# Determinants of Exercise Capacity Following ST-Elevation Myocardial Infarction (STEMI)

**DOI:** 10.3390/jcdd8110140

**Published:** 2021-10-28

**Authors:** Harry Klimis, Aaisha Ferkh, Paula Brown, Robert Zecchin, Mikhail Altman, Liza Thomas

**Affiliations:** 1 Faculty of Medicine and Health, The University of Sydney, Camperdown, NSW 2006, Australia; harry.klimis@sydney.edu.au (H.K.); aaisha_Ferkh@hotmail.com (A.F.); altmanmikhail@hotmail.com (M.A.); 2 Department of Cardiology Westmead Hospital, Westmead, Sydney, NSW 2145, Australia; paula.Brown@health.nsw.gov.au (P.B.); Robert.Zecchin@health.nsw.gov.au (R.Z.); 3 South Western Sydney Clinical School, University of New South Wales, Liverpool, NSW 2170, Australia

**Keywords:** STEMI, myocardial infarction, diastolic function, systolic function, exercise capacity, metabolic equivalents, exercise stress test, echocardiography, left atrial volume

## Abstract

Background: Abnormal left ventricular systolic and diastolic function and reduced exercise capacity are associated with worse prognosis following ST-elevation myocardial infarction (STEMI). However, evidence is lacking on the determinants of exercise capacity following STEMI. We sought to determine the impact of systolic and diastolic dysfunction on exercise capacity and outcomes following first-ever STEMI. Methods: In a retrospective analysis of 139 consecutive STEMI patients who had a transthoracic echocardiogram following STEMI and completed exercise treadmill testing, the primary outcome was to identify clinical and echocardiographic determinants of exercise capacity, and the secondary outcome was to identify determinants of major adverse cardiac events (MACEs). Results: Mean number of metabolic equivalents (METs > 8) was used as a cut-off. Age, female sex, anterior infarction, abnormal diastolic function, minimum left atrial indexed volume (LAVI_min_) ≥ 18 mL/m^2^, average e’, and E/e’ were associated with METs ≤ 8, but not left ventricular ejection fraction (LVEF). On multivariate analysis, LAVI_min_ (OR 4.3, 95%CI 1.3–14.2; *p* = 0.017), anterior infarction (OR 2.6, 95%CI 1.2–5.9; *p* = 0.022), and abnormal diastolic function (OR 3.73, 95%CI 1.7–8.4; *p* = 0.001) were independent predictors of METs ≤ 8. On Kaplan–Meier analysis, METs ≤ 8 (*p* = 0.01) and abnormal diastolic function (*p* = 0.04) were associated with MACEs (median follow-up 2.3 years). METs ≤ 8 was an independent predictor of MACEs (HR 3.4, 95%CI 1.2–9.8; *p* = 0.02). Conclusions: Following first-ever STEMI, increased LAVI_min_, anterior infarction, and abnormal diastolic function were independent predictors of reduced exercise capacity. Furthermore, reduced exercise capacity was an independent predictor of MACEs. These results highlight important prognostic and therapeutic implications related to abnormal diastolic function in STEMI patients that are distinct from those with LV systolic impairment.

## 1. Introduction

Exercise capacity following acute ST-segment elevation myocardial infarction (STEMI) has been used as a surrogate marker and a predictor of poor outcomes [1,2,3]. Specifically, the role of monitored exercise as part of cardiac rehabilitation after acute myocardial infarction (AMI) has been shown to reduce cardiovascular mortality [4], and its importance is reflected in current guidelines [5]. However, the determinants of reduced exercise capacity following STEMI are not well established.

Although left ventricular ejection fraction (LVEF) is a well-established predictor of mortality after myocardial infarction, LVEF per se does not predict exercise capacity, as reported both in patients after myocardial infarction and in unselected patients [6,7]. Only a few studies have demonstrated that diastolic dysfunction may be a more important determinant of exercise capacity as compared to systolic function following myocardial infarction [6,8]. However, there are sparse data on the impact of diastolic function early after STEMI on exercise capacity. Current evidence on exercise capacity includes largely a heterogeneous postinfarct group that does not focus specifically on acute STEMI patients [1,6,8,9]. These studies often include patients with remote myocardial infarction [6], do not comprise a contemporary cohort [9], and have relatively small sample sizes [1,6,9].

Exercise treadmill testing (ETT) and estimation of metabolic equivalents (METs) are used in cardiac rehabilitation for STEMI patients. METs achieved at ETT have been demonstrated to be an independent predictor of cardiac morbidity and mortality in a variety of unselected patients [2,3,10,11] and as a strong predictor of long-term mortality in unselected men referred for ETT [12].

Our aim was to examine the clinical and echocardiographic determinants of exercise capacity defined by METs achieved by ETT, following first-ever STEMI. We additionally sought to determine the prognostic value of METs achieved for a composite of cardiovascular morbidity and mortality. We hypothesised that measures of abnormal diastolic function may be more significant determinants of reduced exercise capacity as compared with systolic function.

## 2. Materials and Methods

### 2.1. Study Design

This was a retrospective cohort study of consecutive patients with first-ever STEMI, presenting between November 2015 and 2017 to Westmead Hospital, Sydney, Australia, a tertiary referral centre.

### 2.2. Study Participants

Patients were included if fulfilling prespecified eligibility criteria based on having received standard of care for STEMI patients at our centre. Eligible patients were aged ≥18 years, admitted with first-ever STEMI, had a transthoracic echocardiogram (TTE) performed following the incident event, and subsequently attended cardiac rehabilitation at our centre. All included patients had exercise capacity assessed by an ETT. Patients in atrial fibrillation at the time of TTE, with greater than moderate valvular regurgitation, with severe mitral annular calcification, or with previous valve replacement/repair were excluded from the analysis as diastolic function in these groups cannot be accurately graded [13].

### 2.3. Study Objectives

The primary objective was to identify clinical and systolic and diastolic echocardiographic parameters that were determinants of exercise capacity at ETT during cardiac rehabilitation. The secondary objective was to determine the prognostic significance of reduced exercise capacity at ETT with major adverse cardiac events (MACEs), defined as the composite endpoint of cardiovascular hospitalisation for recurrent angina, acute coronary syndrome (ACS), heart failure, arrhythmias, and cardiovascular mortality (death related to cardiovascular causes or sudden death).

### 2.4. Data Collection

Prespecified demographic and clinical data were retrospectively collected from electronic medical records. Data were extracted from New South Wales Health Information Exchange by the WSLHD Clinical Analytics and Performance Unit on 30 July 2019, and outcomes were collected until 29 March 2019 (follow-up duration from hospital STEMI presentation: range 1.3–3.4 years; mean 2.3 years). Prespecified echocardiographic parameters were measured from a comprehensive TTE performed following index presentation with STEMI. All TTEs were reanalysed for this study by an investigator blinded to patient, clinical, and ETT details and outcomes. Overall diastolic function was graded and individual parameters of diastolic function (transmitral E and A velocity, E/A ratio, deceleration time, tissue Doppler e’ velocity, E/e’, and left atrial volumes) were evaluated. Diastolic grade was determined according to 2016 guidelines [13] by two independent cardiologists (LT and MA) blinded to patient details and grouped for analysis into abnormal and normal diastolic function (i.e., based on a combination of individual echocardiographic parameters as defined by 2016 guidelines). Discrepancies in diastolic grading between the two independent cardiologists were resolved by discussion. An average of three consecutive measurements was taken for Doppler assessment of transmitral flow velocity (E, E/A, and deceleration time) and tissue Doppler-derived septal and lateral annular early diastolic velocity (average e’). Simpson’s biplane method was used to measure left ventricular (LV) end-diastolic volume (EDV) and end-systolic volume (ESV) and estimate LVEF. Maximum and minimum left atrial volumes were measured from apical 4 and 2 chamber views by Simpson’s biplane method and indexed to body surface area.

Safety of early ETT in STEMI patients has been previously reported by our cardiac rehabilitation program [14]. Exercise stress tests were performed using a General Electric Stress Analyser and treadmill Case Version 6.51 (GE Healthcare Technologies, Milwaukee, WI, USA). Exercise capacity was determined using METs (metabolic equivalents) achieved during maximal symptom and sign limited ETT performed after STEMI using either Bruce, modified Bruce, or modified Naughton protocols. Criteria for test termination included maximum predicted heart rate achieved, systolic blood pressure > 220 mmHg, diastolic blood pressure > 110 mmHg, ischaemic ST-segment changes (at least 4 mm of ST-segment elevation or depression (horizontal or down-sloping) 80 ms after the J point), chronotropic incompetence, new left or right bundle branch block, tachyarrhythmias, heart block, or patient request [14].

METs were dichotomised according to the mean METs achieved as ≤8 and >8 (mean METs achieved in the total study population: 7.8 (SD 3.1) METs). Age was categorised as ≤59 years and >59 years based on the mean age of the group, and gender was categorised as male and female. Smoking status was divided into three groups: current smoker, former smoker, and never smoked. Left atrial indexed volume was dichotomised based on cut-offs from expert consensus and previous studies: ≥34 mL/m^2^ for maximum [15] and ≥18 mL/m^2^ for minimum left atrial indexed volumes [16,17]. LVEF was dichotomised based on a normal value of ≥52% [15]. Tricuspid regurgitant jet velocity was dichotomised based on a value of ≥2.8 m/s [13]. Territory of infarction was based on 12-lead electrocardiogram (ECG) criteria.

### 2.5. Statistical Analysis

SPSS Statistics version 24.0 for Windows (SPSS Inc. An IBM Company, Chicago, IL, USA) was used for analysis; *p*-values ≤ 0.05 were considered statistically significant. Sample distributions of demographics and clinical characteristics at baseline were examined using descriptive statistics. Continuous and categorical variables were expressed as mean ± SD and number (%) respectively. For univariate analyses, two-sample t-tests for continuous variables or χ^2^ tests of independence for categorical variables were performed to determine associations between METs and clinical and echocardiographic factors. Clinical factors included age, gender, body mass index (BMI), waist circumference, STEMI territory (by ECG criteria), diabetes, smoking status, symptom onset to table time and TIMI III flow (thrombolysis in myocardial infarction), days until cardiac rehabilitation from STEMI, and serum creatinine. Echocardiographic parameters of diastolic function assessed included transmitral parameters (peak E velocity, E/A ratio, and deceleration time), mitral annular tissue Doppler parameters (average e’ velocity and E/e’ ratio), indexed left atrial volume (maximum and minimum), and peak tricuspid regurgitant velocity; overall diastolic function was graded as normal or abnormal as described in Section 2.4. LV systolic function was evaluated by LVEF. Variables found to have *p*-values < 0.05 on univariate analyses were included in the multivariable logistic regression model for exercise capacity. Backward elimination was conducted to select the final predictive model for METs ≤ 8. The criterion for the removal of the candidate predictor variables from the multivariable model was set at *p* < 0.10. Cumulative incidence of MACEs was estimated by the Kaplan–Meier method and compared between groups by the log-rank test. For univariate analysis of MACEs, a Cox regression analysis was performed on the variables included in the final multivariable model for METs ≤ 8, in addition to METs achieved. Variables found to have *p*-values < 0.05 on univariate analyses were included in the multivariable Cox regression model for MACEs.

### 2.6. Ethical Considerations

This study was approved by the Western Sydney Local Health District (WSLHD) Human Research Ethics Committee (AU RED LNR/17/WMEAD/192) and conformed to the provisions of the Declaration of Helsinki. Data from this research were completely deidentified and reported only as combined totals.

## 3. Results

### 3.1. Demographics

One hundred thirty-nine consecutive eligible patients (November 2015–2017) were assessed. Table 1 shows the baseline clinical characteristics of the entire cohort. Mean age was 59.2 (SD 11.7) years (range 32–85 years), and 12/139 (8.6%) were female. Mean time from STEMI to TTE was 3.2 (SD 6.1) days. Mean time from STEMI to ETT was 24.7 (SD 17.5) days, and mean number of METs achieved was 7.8 (SD 3.1) METs. All patients included were in sinus rhythm.

Just over half the patients had an anterior infarction (72/139, 51.8%). Most were treated with percutaneous coronary intervention (PCI) (126/139, 90.6%), 4/139 (2.9%) had coronary artery bypass grafting, and 9/139 (6.5%) received medical therapy only. Most received dual antiplatelet therapy (133/139, 95.7%), 4/139 (2.9%) received single antiplatelet therapy with anticoagulation, and 2/139 (1.4%) received triple therapy (dual antiplatelet therapy and anticoagulation). Time from symptom onset to TIMI III flow was available for 107/139 (77.0%) of patients and was 418.9 (SD 698.6) minutes. TIMI III flow was not achieved in 6/139 (4.3%) of patients, and 26/139 (18.7%) did not have symptom onset recorded. Time from symptom onset to table was recorded for 113/139 (81.3%) patients with a mean of 381.2 (SD 675.8) minutes. Most were on appropriate secondary prevention medication at discharge—137/139 (98.6%) were on statin therapy, 116/139 (83.5%) were on beta blockers, and 94/139 (67.6%) were on angiotensin-converting enzyme (ACE) inhibitors/angiotensin receptor blockers.

LVEF was calculated for all patients (Table 1); mean LVEF was 51.2% (SD 9.8%). Diastolic function was graded for all patients; 62/139 (44.6%) had normal diastolic function, 48/139 (34.5%) grade 1 diastolic dysfunction, 29/139 (20.9%) had grade 2 diastolic dysfunction, and none had grade 3 diastolic dysfunction.

### 3.2. Primary Outcome

METs achieved had a significant inverse correlation with left atrial minimum indexed volume (LAVI_min_) (β −0.085, 95%CI −0.162 to −0.008; *p* = 0.031), left atrial maximum indexed volume (LAVI_max_) (β −0.053, 95%CI −0.103 to −0.004; *p* = 0.035), and E/e’ (β −0.383, 95%CI −0.527 to −0.239; *p* < 0.001). METs achieved had a significant positive correlation with E velocity (β 0.512, 95%CI 0.275 to 0.749; *p* < 0.001). There was no significant correlation with LVEF (β 0.015, 95%CI −0.040 to 0.071; *p* = 0.583). Further analyses were performed to determine independent predictors of reduced exercise capacity (mean METs achieved as the binary cut-off (i.e., ≤8 versus >8 METs)).

Individual parameters of diastolic function including transmitral E and A velocity, E/A ratio, deceleration time, e’ velocities, E/e’, and LA maximum and minimum volume were compared between groups based on METs achieved (Table 2). The number of METs achieved was significantly lower in patients with abnormal diastolic function compared to those with normal diastolic function (6.8 (SD 2.8) vs. 9.0 (SD 3.0) METs; *p* < 0.001). Increasing age, presence of anterior infarction, abnormal diastolic function, and individual diastolic function variables (lower average e’ and higher E/e’) were associated with METs ≤8. Additionally, LAVI_min_ ≥ 18 mL/m^2^ (*p* = 0.01) was associated with METs ≤ 8, but LAVI_max_ > 34 mL/m^2^ (*p* = 0.15) was not. LVEF < 52% was not associated with METs ≤ 8 (*p* = 0.15).

Multivariate logistic regression (Table 3) was performed using age (dichotomised as mean of 59 years), anterior infarction, abnormal diastolic function, LAVI_min_ (dichotomised based on upper limit of normal ≥ 18 mL/m^2^), and average E/e’ (e’ velocity was colinear with E/e’ ratio and not included in the multivariate model). Sex was not included as 91.4% were male. LAVI_min_ (OR 4.3, 95%CI 1.3–14.2; *p* = 0.017), anterior infarction (OR 2.6, 95%CI 1.2–5.9; *p* = 0.022), and abnormal diastolic function (OR 3.73, 95%CI 1.7–8.4; *p* = 0.001) were independent predictors of reduced exercise capacity.

### 3.3. Secondary Outcome

**Figure 1:**
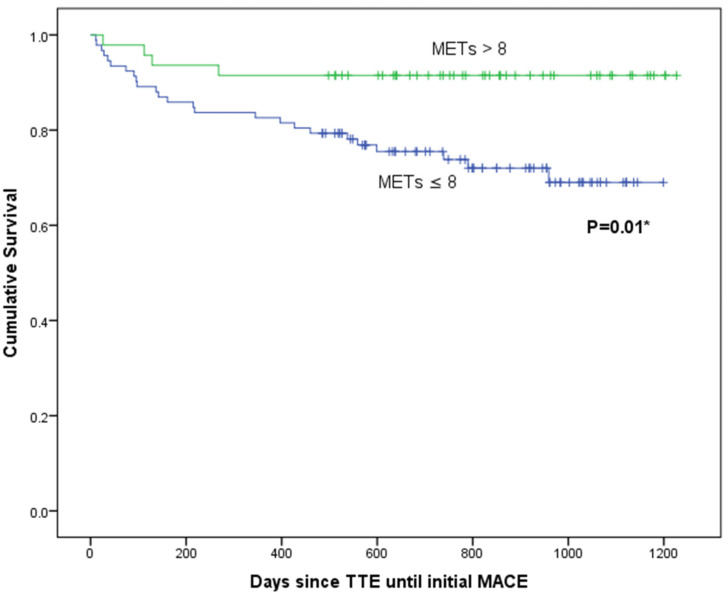
Kaplan–Meier estimates of exercise capacity from time of initial transthoracic echocardiogram to first major adverse cardiac event (MACE). METs, metabolic equivalents; TTE, transthoracic echocardiogram. * Log-rank statistic.

**Figure 2:**
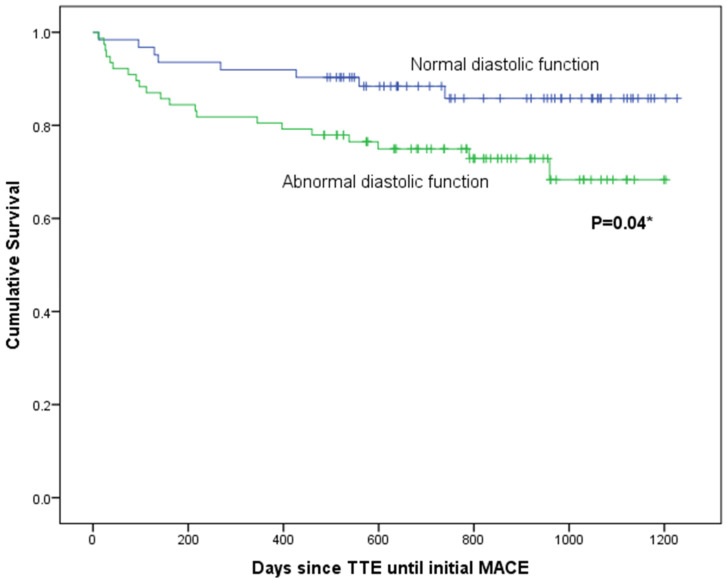
Kaplan–Meier estimates of left ventricular diastolic function (diastolic grade grouped as either normal or abnormal) from time of initial transthoracic echocardiogram to first major adverse cardiac event (MACE). TTE, transthoracic echocardiogram. * Log-rank statistic.

The mean follow-up from TTE was 718 (SD 324) days, and there were 86 cardiac events including 2 deaths (both due to end-stage heart failure) in a total of 29 patients. The diagnoses of first MACE presentation are presented in Table 4. Survival analyses were performed using METs achieved and variables found significant in multivariable logistic regression presented in Table 3. On Kaplan–Meier analysis, METs ≤ 8 was associated with MACEs (log-rank statistic 6.16; *p* = 0.01; Figure 1), as was presence of abnormal diastolic function (log-rank statistic 4.39; *p* = 0.04; Figure 2). There was no association of LAVI_min_ or anterior infarction with MACEs.

On univariate Cox regression analysis, METs ≤ 8 (*p* = 0.02) and abnormal diastolic function (*p* = 0.04) were associated with MACEs. On multivariate analysis (using abnormal diastolic function, METs, anterior infarction, and LAVI_min_), METs ≤ 8 was an independent predictor of MACEs (HR 3.4, 95%CI 1.2–9.8; *p* = 0.02).

## 4. Discussion

The main findings for our study are as follows: (1) Following first-ever STEMI, exercise capacity was reduced in approximately two-thirds of patients to ≤8 METs. (2) LAVI_min_, anterior infarction, and abnormal diastolic function were predictors of METs achieved, independent of age and average E/e’. LV systolic function as determined by LVEF was not a determinant of METs. (3) In this STEMI cohort, a reduced number of METs (<8) was an independent predictor of a composite of nonfatal and fatal MACEs.

Evaluation of diastolic function is complex and not determined by a single parameter. Current guidelines use a combination of several parameters to grade diastolic dysfunction [13]. Diastolic dysfunction is characterised by increased ventricular stiffness that can result in raised LV filling pressures, which are estimated noninvasively using e’ and E/e’, respectively. Chronically elevated LV diastolic pressure in turn causes left atrial enlargement, and recent evidence suggests that LAVI_min_ is often altered before the development of enlarged LAVI_max_ [17]. Previous studies, albeit in a mixed group of patients with ACS, have shown that diastolic function defined by e’ and E/e’ were predictors of exercise capacity, while LVEF was not [1,6,8]. However, in a multivariable model, which included overall diastolic function grade, E/e’ was not an independent predictor, although in a recent study of chronic kidney disease patients without ACS, exercise E/e’ was a predictor of exercise capacity [19]. Similarly, in a large study of a non-ACS population, abnormal diastolic function was an independent predictor of exercise capacity [7]. The strong association of diastolic impairment with exercise capacity can be explained pathophysiologically by the effect of impaired LV compliance and increased filling pressures, limiting the augmentation of stroke volume and cardiac output during exercise [20]. Additionally, both female gender and increasing age have been associated with diastolic impairment [20]. This may in part explain our findings of the association of age and female sex with reduced exercise capacity on univariate analysis and is consistent with a previous large (*n* = 2867) cross-sectional study of patients undergoing exercise echocardiography [7].

Although the prognostic significance of left atrial size and function has been demonstrated in heart failure and other conditions [21,22], data on left atrial size and exercise capacity is sparse. In our study, LAVI_min_ was an independent predictor of exercise capacity. A recent study did demonstrate the effect of left atrial dilatation on exercise capacity [1]; however, to the best of our knowledge, no study has assessed LAVI_min_ as a marker for exercise capacity following STEMI. LAVI_max_ was not associated with exercise capacity, which suggests that LAVI_min_ may be a more sensitive marker of elevation of left ventricular filling pressures. Previous studies have demonstrated that LAVI_min_ is more strongly associated with diastolic dysfunction and increased LV filling pressures than LAVI_max_ [23,24], in part because enlargement of LAVI_min_ would occur prior to an increase in LAVI_max_. As reported recently, enlarged LAVI_min_ with normal LAVI_max_ has intermediate prognostic value, worse than patients with normal LAVI_min_ and LAVI_max_ but improved compared to those with both enlarged LAVI_min_ and LAVI_max_ in patients with myocardial infarction [17].

LV systolic function evaluated by LVEF is a predictor of mortality in STEMI patients [6]. A recent study evaluated LV function recovery following STEMI with serial LVEF measurements three months apart and demonstrated worse outcomes in those with persistently reduced LVEF at follow-up [25]. The utility of advanced echocardiography techniques including global longitudinal strain, a measure of LV contractility, has demonstrated prognostic value following STEMI [26]. However, strain measurements require two-dimensional data to be acquired at high frame rates with specialised software for their measurement, and thus they are not accessible in all centres.

Exercise capacity has been previously correlated to early complete revascularisation in STEMI patients [27]; however, this was not a focus of the current study. Although in our study participants who had lower METs had a longer time to reperfusion, this failed to reach statistical significance, which suggests that ischaemia duration may have little effect on diastolic function, and is consistent with recently published data [28]. Protocolisation of STEMI management [5] and contemporary PCI have led to improvements in reperfusion times making LV systolic impairment less common. In our study, anterior infarction was independently associated with reduced exercise capacity, and this may be related to larger myocardial area at risk and consequent increase in diastolic dysfunction and elevated LV filling pressures [28].

Our study showed reduced exercise capacity to be an independent predictor of MACEs. This is consistent with other studies where the number of METs achieved was an independent predictor of cardiac morbidity and mortality in various clinical settings [3,10,12], including those with recent AMI or after PCI [10], and in STEMI patients [2,11]. However, although diastolic dysfunction was significantly associated with MACEs, this was not an independent predictor in a multivariable model. This may be explained by the relatively small sample size of our study and the absence of subjects with grade 3 diastolic dysfunction (restrictive filling) in our cohort. Restrictive filling pattern has previously been shown to be an independent predictor of cardiac and all-cause mortality in an individual patient meta-analysis [29]. Naqvi et al. [30] demonstrated in patients with first-ever STEMI treated with primary PCI that E/e’ was an independent predictor of in-hospital cardiac events in the short term and superior to all other parameters of LV diastolic and systolic function. However, mean E/e’ was higher in their patient group when compared to our study population, suggesting more advanced diastolic dysfunction. Additionally, our follow-up duration was much longer, resulting in differing durations of medical treatments.

This study has some limitations. Firstly, this was a single-centre retrospective study with a relatively small number of patients and low event rates. Nonetheless, our results are novel and clinically important as a ‘proof of concept study’ and merit further investigation with larger studies. Secondly, peak troponin as a biochemical marker of infarct size was not assessed as the pathology testing at our hospital capped measurement of high-sensitive troponin I at 40,000 ng/L, preventing assessment of peak levels. Thirdly, details on pre-existing diastolic dysfunction are lacking to determine whether diastolic dysfunction was present prior to STEMI or developed after STEMI. However, determining pre-existing diastolic function would not be possible given this was a cohort of first-ever STEMI of which the timing is unpredictable. We also did not perform analysis of patient medications given the relatively high use of appropriate therapy. Fourthly, this was a retrospective study, and hence advanced echocardiographic measurements such as strain analysis could not be evaluated. Finally, <10% of our STEMI study population was female, which is lower compared to Australian registry data (25% female) [31] and thus not generalisable to females. This may reflect a degree of selection bias in our study (i.e., patients who survived STEMI, had a TTE, attended cardiac rehabilitation, and completed ETT).

## 5. Conclusions

In patients who underwent ETT following first-ever STEMI, LAVI_min_, anterior infarction, and abnormal diastolic function were independent predictors of exercise capacity and superior to clinical variables and other echocardiographic parameters including LVEF, demonstrating the impact of abnormal diastolic function on exercise capacity in STEMI patients. Furthermore, reduced exercise capacity was an independent predictor of MACEs, demonstrating the prognostic value of this relatively easy to obtain parameter in STEMI patients undergoing cardiac rehabilitation. Although our data require confirmation in larger prospective studies, the results highlight important implications for monitoring and surveillance of STEMI patients with diastolic dysfunction distinct from those with LV systolic impairment.

## Figures and Tables

**Table 1:** Baseline clinical characteristics.

Variable	Cohort (*n* = 139)
Age; mean ± SD (years)	59.2 ± 11.7
Male; *n* (%)	127 (91.4%)
BMI; mean ± SD (kg/m^2^)	27.7 ± 4.7
Waist circumference; mean ± SD (cm)	99.2 ± 11.5
Current smokers; *n* (%)	37 (26.6%)
Former smokers; *n* (%)	29 (20.9%)
Never smoked; *n* (%)	73 (52.5%)
Hypertension; *n* (%)	63 (45.3%)
Hypercholesterolaemia; *n* (%)	134 (96.4%)
Diabetes mellitus; *n* (%)	36 (25.9%)
Family history of premature CAD; *n* (%)	52 (37.4%)
Creatinine; mean ± SD (µmol/L)	84.3 ± 29.5
Anterior infarction; *n* (%)	72 (51.8%)
Inferior infarction; *n* (%)	61 (43.9%)
Lateral infarction; *n* (%)	6 (4.3%)
Normal diastolic function	62 (44.6%)
Grade 1 diastolic dysfunction	48 (34.5%)
Grade 2 diastolic dysfunction	29 (20.9%)
Grade 3 diastolic dysfunction	0 (0%)
Normal LV systolic function (LVEF * 52–72%); *n* (%)	67 (48.2%)
Mild LV systolic impairment (LVEF * 41–51%); *n* (%)	51(36.7%)
Moderate LV systolic impairment (LVEF * 30–40%); *n* (%)	17 (12.2%)
Severe LV systolic impairment (LVEF * <30%); *n* (%)	4 (2.9%)

SD, standard deviation; BMI, body mass index; CAD, coronary artery disease; LV, left ventricular. * 2DE-derived LVEF as calculated by Simpson’s biplane [18].

**Table 2:** Baseline variables of interest by METs achieved in a univariate logistic regression.

	METs Achieved		*p*-Value
	≤8 METs	>8 METs	
Age > 59 years; *n* (%)	51 (55.4)	12 (25.5)	0.001
Male; *n* (%)	81 (88.0)	46 (97.9)	0.051
BMI (kg/m^2^); mean (SD)	27.3 (5.1)	28.4 (4.0)	0.221
Waist circumference (cm); mean (SD)	98.7 (11.9)	100.1 (10.8)	0.510
Diabetes; *n* (%)	27 (29.3)	9 (19.1)	0.194
Hypertension; *n* (%)	45 (48.9)	18 (38.3)	0.237
Smoking status			0.374
Never smoked; *n* (%)	48 (52.2)	25 (53.2)	
Former smoker; *n* (%)	22 (23.9)	7 (14.9)	
Current smoker; *n* (%)	22 (23.9)	15 (31.9)	
Time from pain to table (min); mean (SD)	420.5 (733.0)	309.3 (558.0)	0.084
Pain to TIMI III (min); mean (SD)	468.4 (757.3)	346.1 (565.3)	0.382
Days until cardiac rehabilitation; mean (SD)	22.3 (12.9)	29.4 (23.7)	0.347
Creatinine (μmol/L); mean (SD)	84.0 (33.9)	84.8 (18.4)	0.246
Peak E (cm/s); mean (SD)	70.7 (18.7)	70.4 (13.6)	0.845
Peak A (cm/s); mean (SD)	76.0 (20.2)	69.0 (18.3)	0.068
E/A; mean (SD)	0.98 (0.32)	1.09 (0.34)	0.060
DT (ms); mean (SD)	193.7 (52.8)	193.6 (40.4)	0.608
e’ septal (cm/s); mean (SD)	5.8 (1.6)	7.1 (1.9)	<0.001
e’ lateral (cm/s); mean (SD)	7.4 (2.6)	9.2 (2.6)	<0.001
Average e’ (cm/s); mean (SD)	6.6 (2.0)	8.1 (2.0)	<0.001
E/e’; mean (SD)	11.3 (3.5)	9.4 (2.8)	0.001
LA volume indexed (maximum) > 34 mL/m^2^; *n* (%)	45 (48.9)	17 (36.2)	0.153
LA volume indexed (minimum) ≥ 18 mL/m^2^; *n* (%)	25 (27.2)	4 (8.5)	0.009
LVEF < 52%; *n* (%)	55 (59.8)	22 (46.8)	0.145
Peak TR vel > 2.8 m/s; *n* (%)	10 (10.9)	4 (8.5)	0.662
Diastolic dysfunction *; *n* (%)	62 (67.4)	15 (31.9)	<0.001

BMI, body mass index; TIMI, thrombolysis in myocardial infarction (TIMI III refers to complete perfusion); DT, deceleration time; LA, left atrium; LVEF, left ventricular ejection fraction; TR, tricuspid regurgitation. * Grouped as absent or present.

**Table 3:** Multivariable logistic regression for METs ≤ 8: final model.

Variable	Odds Ratio (95% Confidence Interval)
Left atrial indexed volume (minimum)	OR 4.3 (1.3–14.2) *
Abnormal diastolic function	OR 3.7 (1.7–8.4) **
Anterior infarction	OR 2.6 (1.2–5.9) *

* *p* < 0.05; ** *p* < 0.01.

**Table 4:** First major adverse cardiac event diagnoses.

Diagnosis	Frequency	*n* = 139 (%)
Recurrent angina	7	5.0
Unstable angina	5	3.6
NSTEMI	9	6.5
STEMI	1	0.7
Heart failure	4	2.9
Arrhythmia	3	2.2

ACS, acute coronary syndrome; NSTEMI, non-ST-elevation myocardial infarction; STEMI, ST-elevation myocardial infarction.

## Data Availability

In line with the requirements of the ethics committee who approved this research, requests for access to data are to be made in writing to the corresponding author. Only deidentified participant data can be made available to suitably qualified researchers on reasonable request.

## Abbreviations

ACE	Angiotensin-converting enzyme
ACS	Acute coronary syndrome
AMI	Acute myocardial infarction
ECG	Electrocardiogram
EDV	End-diastolic volume
ESV	End-systolic volume
ETT	Exercise treadmill testing
LAVI_max_	Maximum left atrial indexed volume
LAVI_min_	Minimum left atrial indexed volume
LV	Left ventricular
LVEF	Left ventricular ejection fraction
MACEs	Major adverse cardiac events
METs	Metabolic equivalents
NSTEMI	Non-ST-elevation myocardial infarction
PCI	Percutaneous coronary intervention
STEMI	ST-elevation myocardial infarction
TTE	Transthoracic echocardiogram
